# The role of generative AI tools in shaping mechanical engineering education from an undergraduate perspective

**DOI:** 10.1038/s41598-025-93871-z

**Published:** 2025-03-17

**Authors:** Harshal Akolekar, Piyush Jhamnani, Vikash Kumar, Vinay Tailor, Aditya Pote, Ankit Meena, Kamal Kumar, Jagat Sesh Challa, Dhruv Kumar

**Affiliations:** 1 Department of Mechanical Engineering, Indian Institute of Technology, Jodhpur, 342030 India; 2 School of AI & Data Science, Indian Institute of Technology, Jodhpur, 342030 India; 3 Department of Electrical Engineering, Indian Institute of Technology, Jodhpur, 342030 India; 4 Department of Computer Science & Information Systems, Birla Institute of Technology & Science, Pilani, 333031 India; 5 Department of Computer Science and Engineering, Indraprastha Institute of Information Technology, 110020 Delhi, India

**Keywords:** Generative AI, ChatGPT, Copilot, Gemini, Mechanical engineering, Mechanical engineering, Computer science

## Abstract

This study evaluates the effectiveness of three leading generative AI tools-ChatGPT, Gemini, and Copilot-in undergraduate mechanical engineering education using a mixed-methods approach. The performance of these tools was assessed on 800 questions spanning seven core subjects, covering multiple-choice, numerical, and theory-based formats. While all three AI tools demonstrated strong performance in theory-based questions, they struggled with numerical problem-solving, particularly in areas requiring deep conceptual understanding and complex calculations. Among them, Copilot achieved the highest accuracy (60.38%), followed by Gemini (57.13%) and ChatGPT (46.63%). To complement these findings, a survey of 172 students and interviews with 20 participants provided insights into user experiences, challenges, and perceptions of AI in academic settings. Thematic analysis revealed concerns regarding AI’s reliability in numerical tasks and its potential impact on students’ problem-solving abilities. Based on these results, this study offers strategic recommendations for integrating AI into mechanical engineering curricula, ensuring its responsible use to enhance learning without fostering dependency. Additionally, we propose instructional strategies to help educators adapt assessment methods in the era of AI-assisted learning. These findings contribute to the broader discussion on AI’s role in engineering education and its implications for future learning methodologies.

## Introduction

With the rise of artificial intelligence (AI), the world stands on the precipice of an unprecedented technological revolution. Over the last few decades, AI has transitioned from the pages of science fiction to virtually penetrating every aspect of our lives. This profound transformation is not merely a technological advancement, it marks a paradigm shift in how we interact with the world around us, process information and solve problems. At its root, AI involves the creation of intelligent machines that mimic human cognitive functions such as reasoning, learning, problem-solving, and decision-making. What sets this revolution apart is the rapid pace at which AI has progressed^1,2^. It has transcended its role as a niche concept to permeate numerous aspects of our lives, from virtual personal assistants in our smartphones to complex algorithms that power autonomous vehicles, intricate financial models, and now even education. The latest revolution in AI is the creation of generative AI tools, which the common man can access at his fingertips. Several generative AI tools are gaining popularity - the major ones being ChatGPT^3^ (based on the Generative Pre-trained Transformer^4^) of OpenAI, Gemini^5^ of Google, and Copilot^6^ of Microsoft. They use deep learning and natural language processing techniques to generate human-like responses based on the context of conversations. They are capable of handling diverse language tasks, from answering questions and generating text to supporting coding and content creation. Since their introduction, significant research has examined the advantages and disadvantages of these tools, especially in everyday applications such as education.

The rapid spread of generative AI tools has generated significant debate among educators globally^7–15^. Some view it as a beneficial learning tool, while others worry it may lead to cheating and misinformation. Futterer et al.’s^16^ analysis of over 16 million tweets reveals mixed sentiments, with education being the most discussed topic, highlighting differing perspectives between public opinion and academic views. In the context of higher education, Cotton et al.^8^ argue that generative AI tools offer benefits like asynchronous learning, timely feedback, supporting student collaboration, and facilitating remote learning. However, they caution that these tools can enable cheating and make it challenging to differentiate between human and AI-generated content. Nikolic et al.^17^ explored the use of ChatGPT for solving engineering assessments in seven Australian universities. They found that ChatGPT excelled in some assessment types such as quizzes, and short answers but was not too effective for image-based questions, oral presentation assessments, and numericals. Lund and Wang^18^ discussed the impact of ChatGPT in improving research in academia as it can assist in the literature review, text generation and question answering, and data analysis. Berdanier and Alley^19^ state that tools like ChatGPT should not be seen as a replacement for technical writing and that engineers still need to be taught ‘how to write’. They correlate the writing ability of a person with their ability to think. Qadir et al.^20^, in the realm of engineering education, found that ChatGPT enhances personalized learning with customized feedback and simulations but may perpetuate biases, spread misinformation, and raise ethical concerns, including potential misuse and job displacement. Extensive research^21–28^ has focused on the application of generative AI tools in computer science and engineering education, driven by the increasing demand for these fields. Joshi et al.^21^ analyzed the strengths and weaknesses of ChatGPT for solving assignments and exam questions of undergraduate computer science students. The evaluation highlights the potential consequences of students excessively relying on ChatGPT for completing assignments and exams, including the risk of self-sabotage. This dependence could undermine their learning and critical thinking skills, leading to long-term academic disadvantages. Using a mixed-methods approach,^29^ Budhiraja et al.^22^ used student surveys and interviews to assess the benefits and challenges of ChatGPT. Over 57% of students viewed ChatGPT positively for coursework support. Balart and Shryock^30^ explore the impact of ChatGPT in enhancing learning and skill development for first-year computer programming students, comparing its effectiveness against traditional teaching assistants and combined methods in programming courses, while also addressing AI’s benefits, challenges, and ethical implications with a mixed methods approach.

Several studies also deal with the use of generative AI tools in other areas of engineering education^31–35^. Frenkel and Emara^33^ compared the performance of the free ChatGPT 3.5 and paid ChatGPT 4.0 variants by asking about 50 questions from the Fundamental of Engineering exam. ChatGPT 4.0 got 76% of the questions correct, whereas ChatGPT 3.5 could only manage 51% correct. Tiro^36^ investigated the application of ChatGPT to a few mechanical engineering problems. The study concluded that ChatGPT is not recommended for solving mechanical engineering calculations due to its tendency to produce incorrect solutions, which could be misleading and potentially dangerous in real-world applications. Tsai et al.^37^ explored the use of ChatGPT for solving chemical engineering problems, such as steam power cycles. Students were initially required to find the correct solutions analytically. With the aid of ChatGPT, they were then able to engage in deeper problem analysis. However, since ChatGPT made errors in solving the problems, this process helped students critically evaluate the AI’s responses. Uddin et al.^38^ examined the impact of ChatGPT on civil engineering students’ assignments, finding that it enabled them to produce more comprehensive and detailed responses. The tool facilitated deeper engagement with the material, helping students generate more informative content for their coursework. Recent advancements in generative AI offer new possibilities for engineering education, but their effectiveness varies. Few-shot identification in stochastic systems^39^ highlights adaptive learning’s role in improving AI decision-making, while deep learning in education^40^ emphasizes the need for structured AI integration. Multihead attention self-supervised models^41^ suggest that enhanced contextual processing could improve AI accuracy. Similarly, AI applications in digital twin systems and network modeling^42^ highlight the need for reliable AI-generated insights, a challenge also present in engineering education. Deep transfer learning for city-scale traffic generation^43^ could improve AI’s numerical problem-solving capabilities, while research on scalable digital twin systems^44^ reinforces the need for structured AI integration in engineering education. These studies collectively support AI as a complementary tool rather than a replacement for traditional problem-solving.

A few studies also discuss the differences between various generative AI models, primarily between ChatGPT, Copilot, and Gemini. Ashwal et al.^45^ compared the accuracy of ChatGPT, BingAI (now Copilot), and Bard (now Gemini) to predict drug-drug interactions. They found that ChatGPT had the lowest accuracy rate of 0.469, and BingAI the highest with 0.788. Rudolph et al.^46^ analyzed ChatGPT, BingAI, and Bard on multi-disciplinary tests relevant to higher education and found that BingAI and Bard saved students from getting an ‘F-grade’, which indicates that none of the generative AI tools performed up to expectation, with ChatGPT performing the worst. Nikolic et al.^17^ conducted a multi-disciplinary study evaluating the performance of generative AI tools like ChatGPT-4 and Gemini across 10 engineering subjects. They created an opportunity matrix highlighting the strengths and weaknesses of each tool. Overall, ChatGPT-4 was found to be a good all-round option. Rossettini et al.^47^ investigated the use of ChatGPT-4, Copilot, and Gemini on Italian entrance tests for healthcare science degrees by asking them 820 multiple-choice questions (MCQs). They found that ChatGPT-4 and Copilot performed better than Gemini.

While numerous studies explore the various applications of generative AI in engineering education, a significant gap remains in assessing the performance of different generative AI tools specifically within mechanical engineering subjects, for a large variety of question types. More targeted research is needed to address this subject-specific evaluation across a large number of questions and question types. Additionally, there is a need to understand the mindset of mechanical engineering students and instructors in adapting to generative AI tools. This study evaluates the efficacy of three leading generative AI tools-ChatGPT, Gemini, and Copilot-in solving a wide array of undergraduate mechanical engineering problems. The research involves a comprehensive analysis of these tools’ performance on 800 questions from seven subjects sourced from leading global universities and examinations, aiming to assess their accuracy, reliability, and potential applications in mechanical engineering education. The student perception of generative AI tools is also analyzed in terms of 172 survey responses and 20 interviews. These qualitative methods shed light on how mechanical engineering students make use of generative A tools. Furthermore, our study explores the challenges instructors may encounter when assessing students and offers strategies for overcoming them. Based on our findings, we present recommendations for future design and development of mechanical engineering educational tools built on Large Language Models (LLMs), ensuring more effective and reliable academic evaluations. Our research focused on three key research questions.

- RQ1: What is the accuracy of generative AI tools in answering undergraduate mechanical engineering questions?
- RQ2: What is the overall perception of generative AI tools amongst undergraduate mechanical engineering students?
- RQ3: How can mechanical engineering students and instructors constructively make use of generative AI tools to enhance the overall learning and teaching experience?

## Methods and analysis

In order to analyze the impact of generative AI tools in engineering education, it is best to use research frameworks^48^. Methodological frameworks^49–51^ such as qualitative, quantitative, or mixed-methods and analytical frameworks^52,53^ such as thematic analysis are quite prominent. A mixed-methods research methodology^49^ with an exploratory design was used in this study. The performance of ChatGPT, Google Gemini, and Microsoft CoPilot was quantified by asking them numerous questions related to mechanical engineering. Additionally, we collect a quantitative evaluation of these tools with a survey of undergraduate mechanical engineering. Interviews were also conducted to understand the students’ perception of these tools qualitatively.

### Questions

To evaluate the performance of these AI tools, we used 800 questions from seven foundational subjects of undergraduate mechanical engineering: thermodynamics, fluid mechanics, strength of materials, machine design, manufacturing processes, engineering mechanics, and heat transfer. These questions and their solutions were sourced from world-renowned universities such as MIT, Stanford, and the Indian Institutes of Technology (IITs), as well as from the Graduate Aptitude Test in Engineering (GATE), a prestigious national-level examination in India. GATE scores are widely recognized for admission into postgraduate programs and employment in public sector organizations, with some universities in Germany and Singapore also accepting them^54^. Given the large participation of undergraduate and graduate students in the GATE exam annually, we integrated this factor into our assessment framework.

The dataset covered three question formats: multiple-choice questions (MCQs) with a single correct answer from four or five options, numerical problems requiring stepwise calculations, and theory-based questions demanding descriptive responses. The question selection process prioritized curriculum alignment, cognitive complexity, and diversity to ensure a comprehensive evaluation. A validation process was conducted to refine the database in which a panel of three faculty members reviewed the questions for clarity, relevance, and difficulty, making necessary modifications. To prevent bias toward specific AI tools, we ensured a balanced mix of text-based and numerical problems. Testing was performed under controlled conditions, using the latest publicly available versions of ChatGPT, Gemini, and Copilot, without manual corrections or iterative attempts. Responses were evaluated through a structured grading framework: MCQs were matched against a predefined answer key, numerical solutions were checked for accuracy and stepwise correctness, and theory-based answers were graded by faculty using a standardized rubric. Additionally, each AI tool was tested two times on the full dataset to analyze variations and detect hallucinations, computational errors, or inconsistencies. This rigorous selection, validation, and testing methodology ensures that the assessment of AI performance remains accurate, unbiased, and reproducible. In evaluating responses to the questions, the following assumptions and considerations were taken into account:

- MCQs and Numerical Questions: Responses were classified as correct or incorrect based on final answer accuracy. For GATE numerical questions, minor variations were allowed within the acceptable range to reflect practical engineering problem-solving.
- Multi-Quantity Numerical Questions: A response was deemed correct if more than half of the calculated quantities matched expected results, ensuring a balanced evaluation of accuracy.
- Theory Questions: Answers were evaluated on relevance and context. Responses deviating from the topic or failing to address key points were marked incorrect.
- AI Tool Versions: Only the latest free versions (e.g., ChatGPT-4o mini) were used, as students predominantly rely on unpaid tools. Questions excluded images, as paid versions offer unrestricted image-based responses.
- Single Attempt Policy: Each question was posed once without follow-up prompts, ensuring a fair and consistent assessment of AI performance.

### Survey demography

We conducted a survey using Google Forms, which was distributed via authorized personnel through mailing lists at two top-ranked engineering universities in India - Indian Institute of Technology Jodhpur (IITJ) and Birla Institute of Technology and Science (BITS) Pilani. The survey email explained the study’s purpose and asked students to participate by responding to the form. The survey focused on various aspects of generative AI tools within an academic context for undergraduate mechanical engineering students. The study included thirteen questions that addressed various aspects of the participant’s experiences and perceptions. The questions focused on the frequency of use and popular use cases. Some questions explored the participants’ overall perspectives on the benefits and drawbacks of generative AI tools, as well as their thoughts on the utility of generative AI in mechanical engineering education. This comprehensive approach aimed to gather insights into both the practical applications and perceived limitations of these tools in an academic context. These themes were explored to establish a foundation for understanding typical student perceptions, attitudes, challenges, and expectations related to ChatGPT. The survey employed a variety of question formats, including single-choice questions, Likert scale questions, and MCQs. The survey questions may be found in Appendix A. Additionally, it included two text-based response fields, allowing us to gather both qualitative and quantitative insights. Completing the survey typically took participants between 3 and 5 min.

There were 172 survey respondents 65.7% were from IIT-J and 34.3% were from BITS. 6.4% students just graduated, 30.81% were in their final (fourth) year, 30.23% were in their third year, and 32.56% were in their second year. First-year students do not have core mechanical engineering subjects and hence were not asked to participate in the survey. The majority of the students (78.5%) were pursuing undergraduate degrees in mechanical engineering, with 14.5% from manufacturing engineering and 7% from materials engineering. These programs are considered subsets of mechanical engineering and share many common courses with it. This overlap highlights the interconnected nature of these disciplines and emphasizes the foundational role of mechanical engineering in related fields.

### Interview demography

20 interviews of undergraduate mechanical engineering students from IIT-J and BITS were conducted. Participants for the interviews were recruited through snowball sampling^55^. Interviews were conducted via Google Meet, and audio recordings were made for subsequent analysis. Both written and verbal consent were obtained from the interviewees for their participation and the recording of the interviews. The interviews aimed to comprehend undergraduate mechanical engineering students’ academic experiences with various generative AI tools in several scenarios such as assignments, autodidacticism (self-teaching), content generation, preparation for interviews, and much more. The questions aimed to get deeper insights into the perceptions of undergraduate mechanical engineering students and understand their motivations, biases, habits and challenges in using generative AI tools. A total of six questions were incorporated into the interview structure, with clear protocols and guidelines in place. The interview questions are listed in Appendix B. These covered the interview process, the objective of each question (e.g., awareness, familiarity, challenges), and optional probing questions to ensure depth while maintaining flexibility. This structure enabled the research team to explore insights dynamically, keeping a consistent approach across participants. Interviewees were recruited from the two universities and academic years to capture different perspectives. No specific academic performance criteria were applied beyond being enrolled in or just graduated from an undergraduate mechanical engineering program. The interviews were transcribed by the research team. and semantic coding^56^ of the transcripts was undertaken. These codes were grouped into intermediate themes. A subsequent latent coding phase refined these themes, leading to the final set of overarching themes resulting in thematic analysis^52^.

### Ethical considerations

Throughout the study, we carefully addressed ethical concerns for transparency and participant privacy. Our materials and procedures were approved by the university’s Institutional Review Board and were carried out in accordance with relevant guidelines and regulations. In surveys, we highlighted voluntary participation, response confidentiality, and the study’s purpose. The survey introduction also explained the participants’ role in contributing to the study and assured them of the anonymity of their responses, as no names or identifying information was collected. Before interviews, participants provided explicit written and verbal informed consent, assuring anonymity. Thus informed consent was obtained from all subjects. Data was anonymized, and securely stored with limited team access. Our research team comprises individuals with expertise in human-computer interaction (HCI), artificial intelligence, and engineering education.

## Results

This section highlights the key findings by analyzing the responses to the questions asked to the generative AI tools. A quantitative evaluation in terms of a survey and a qualitative evaluation of the perception of undergraduate mechanical engineering students are also presented.

### Question analysis

This section discusses the subject-wise and category-wise accuracy of the 800 questions asked of the three generative AI tools. Tables 1, 2 and 3 show the detailed breakdown of category-wise and subject-wise (SW) accuracy for the responses from ChatGPT, Copilot, and Gemini respectively. 397 MCQs, 271 numericals, and 132 theory-based questions were asked to these tools. 168 questions each were asked from fluid mechanics (FM), 167 from thermodynamics (TD) (including applied thermodynamics), and 165 from heat transfer (HT). 100 questions each were asked from production engineering (PE) and manufacturing processes (MP) and 50 each were asked from kinematics & dynamics of machines (KD) and statistical mechanics (SM). The subjects of fluid mechanics, thermodynamics, and heat transfer contain a lot of text-based numericals, and hence we asked more numerical-based questions than the other four subjects. Almost all questions from kinematics and dynamics are image-based and hence we could only find one non-image-based numerical of adequate difficulty that was answered correctly by all the AI tools. Statistical mechanics numerical questions are also highly image-based and hence we posed only 5 numerical questions to each of the AI tools. Since these two subjects have a greater number of theory-based questions, they had a higher subject-wise accuracy score across all AI tools.

**Table 1:** Subject and question-category breakdown and accuracy measure using ChatGPT (C: Correct, T: Total, A: Accuracy (%)).

	MCQ			Numerical			Theory			SW accuracy (%)
Subject	C	T	A	C	T	A	C	T	A	
Fluid mechanics	38	75	50.61	15	75	20.00	15	18	83.33	40.48
Thermodynamics	39	75	52.00	14	75	18.67	14	17	82.35	40.12
Heat transfer	44	75	58.67	20	75	26.67	13	15	86.66	46.66
Production engineering	27	57	47.37	8	21	38.10	18	22	81.81	53.00
Manufacturing processes	25	60	41.67	2	19	10.52	18	21	85.71	45.00
Kinematics & dynamics of machines	12	30	40.00	1	1	100	15	19	78.95	56.00
Statistical mechanics	14	25	56.00	4	5	80.00	17	20	85.00	70.00
Category-wise accuracy (%)			50.13			23.62			83.33	46.63

**Table 2:** Subject and question-category breakdown and accuracy measure using Copilot.

Subject	MCQ			Numerical			Theory			SW accuracy (%)
	C	T	A	C	T	A	C	T	A	
Fluid mechanics	46	75	61.33	29	75	38.67	15	18	83.33	53.57
Thermodynamics	51	75	68.00	25	75	33.33	14	17	82.35	53.89
Heat transfer	53	75	70.67	28	75	37.33	14	15	93.33	57.58
Production engineering	38	57	66.67	9	21	42.86	19	22	86.36	66.00
Manufacturing processes	33	60	55.00	7	19	36.84	19	21	90.48	59.00
Kinematics & dynamics of machines	21	30	70.00	1	1	100.00	19	19	100.00	82.00
Statistical mechanics	20	25	80.00	5	5	100.00	17	20	85.00	84.00
Category-wise accuracy (%)			65.99			38.38			88.64	60.38

**Table 3:** Subject and question-category breakdown and accuracy measure using Gemini.

Subject	MCQ			Numerical			Theory			SW accuracy (%)
	C	T	A	C	T	A	C	T	A	
Fluid mechanics	43	75	57.33	24	75	32.00	16	18	88.89	49.40
Thermodynamics	55	75	73.33	23	75	30.67	15	17	88.24	55.69
Heat transfer	53	75	70.67	27	75	36.00	13	15	86.67	56.36
Production engineering	33	57	57.89	9	21	42.86	19	22	86.36	61.00
Manufacturing processes	30	60	50.00	5	19	26.32	20	21	95.24	55.00
Kinematics & dynamics of machines	18	30	60.00	1	1	100.00	16	19	84.21	70.00
Statistical mechanics	16	25	64.00	3	5	60.00	18	20	90.00	74.00
Category-wise accuracy (%)			62.47			33.95			88.64	57.13

**Fig. 1:**
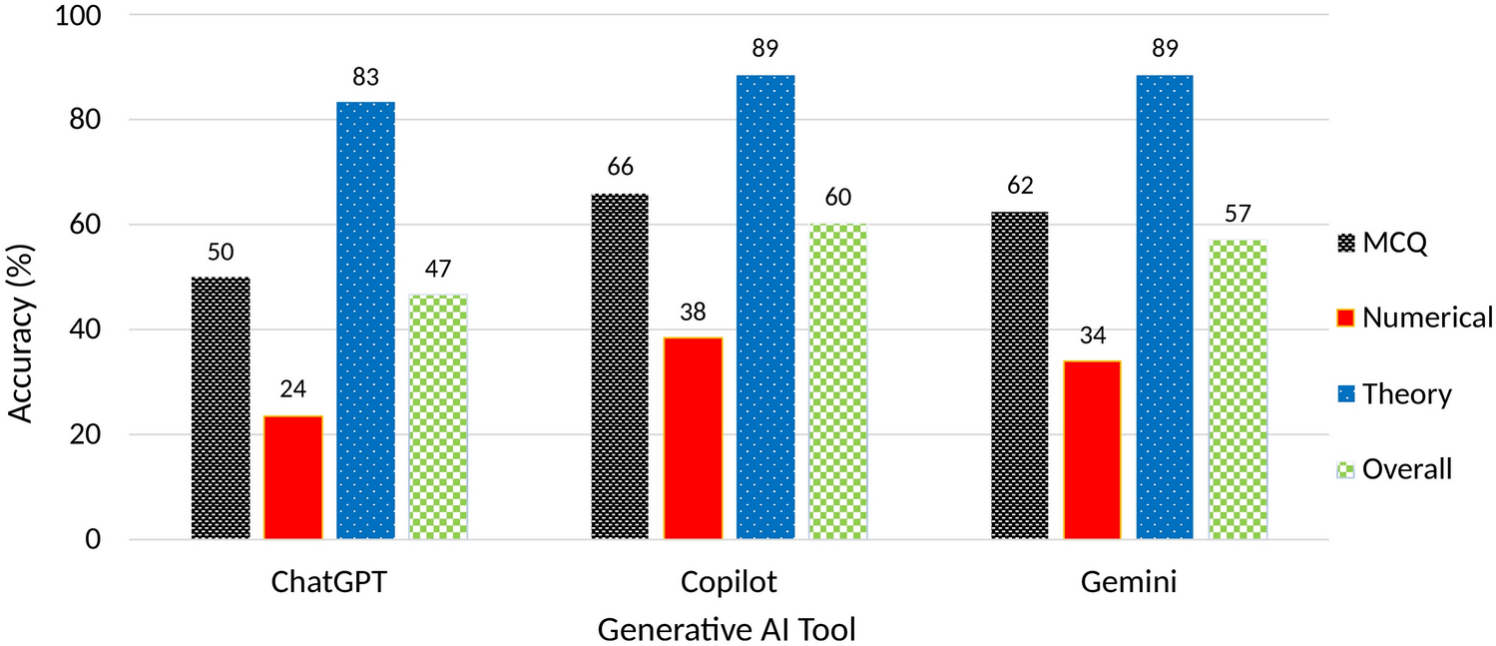
Category-wise accuracy of generative AI tools in answering mechanical engineering questions.

ChatGPT was able to answer 46.63% of the total questions correctly, Gemini 57.13%, and Copilot gave the best performance of 60.38%. All the tools were able to answer theory questions quite accurately and the mean accuracy was above 80%, as these AI tools have been developed to answer theory or information-based questions. Copilot and Gemini got nearly 2/3rd of MCQ questions correct whereas ChatGPT only got half of them correct. All the AI tools performed poorly for numerical questions, with Copilot and Gemini answering about a third of the questions correctly and ChatGPT only about a quarter. Figure 1 shows the category-wise and overall accuracy (rounded to the nearest integer) of the AI tools in answering questions.

**Fig. 2:**
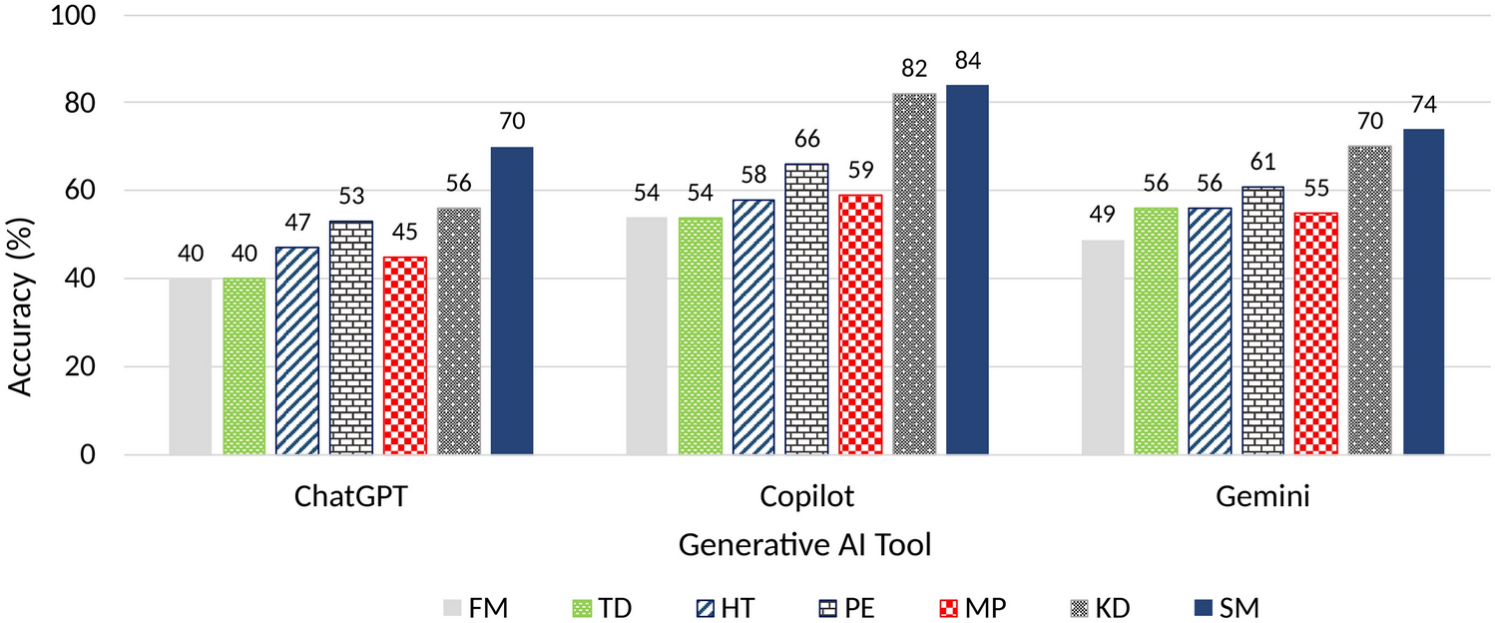
Subject-wise accuracy of generative AI tools in answering mechanical engineering questions.

From Table 1, for ChatGPT, the highest accuracy is observed in theory questions across all subjects, averaging 83.33%. Numerical questions have the lowest category-wise accuracy at 23.62%. The average accuracy for MCQs is around 50.13%. Subjects like heat transfer (58.67%) and thermodynamics (52.00%) outperform others. In terms of subject-wise overall accuracy, statistical mechanics (70.00%) and kinematics & dynamics of machines (56.00%) stand out, while thermodynamics (40.12%) and fluid mechanics (40.48%) have lower scores. From Table 2, for Copilot, the theory-based questions demonstrate the highest accuracy across all subjects (88.64%). Numerical-type questions show the lowest accuracy (38.38%). Multiple-choice questions (MCQs) fall in between, with an accuracy of 65.99%. Numerical questions in fluid mechanics, thermodynamics, and manufacturing processes are notably weaker, with accuracy below 40%. From Table 3, Gemini gave an accuracy of 62.47% in answering MCQs, with the best performance in thermodynamics. As with ChatGPT and Gemini, it performed poorly in numericals, especially that of manufacturing processes with an accuracy of 26.32%. It performed quite well in answering theory-based questions. It was found that Copilot outperformed Gemini and ChatGPT in terms of accuracy across all subjects and categories. Gemini only did slightly better than Copilot in MCQs of thermodynamics and theory questions of production engineering and statistical mechanics. Low scores in numerical questions suggest that the generative AI tools may need refinement in handling complex engineering calculations in these areas. Figure 2 shows the subject-wise accuracy from the three tools, rounded to the nearest integer.

### Survey responses

172 responses for the survey of undergraduate mechanical engineering students, were obtained. Two single-response questions dealt with the duration and frequency of usage of generative AI tools. 78.5% stated that they had been using AI tools for more than 3 months, 14.5% for 2–3 months, 4.1% for 1–2 months, and 2.9% for less than a month. This shows a high retention rate among students. When asked how often they use AI tools, 32.6% of the participants use it daily, 29.1% use it weekly, 32.6% use it occasionally, and 5.8% use it rarely. Additionally, 84.9% of the participants were quite familiar with AI tools and 15.1% said they are somewhat familiar. These results show that the students are actively using AI tools.

**Fig. 3:**
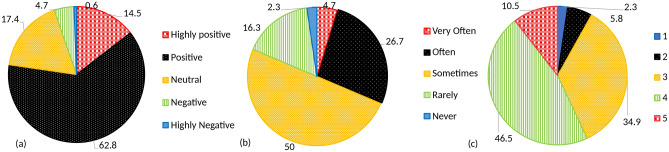
(**a**) Overall perception of students towards AI tools as a useful learning and education tool. (**b**) Frequency of problems while using AI tools. (**c**) On a scale of 1-5, to what extent can AI tools aid in course-work related queries?

When asked regarding the overall perception of students towards AI tools, as useful learning and educational tools, 14.5% were highly positive. The vast majority (62.8%) was positive, 17.4% were neutral while the remaining were negative or highly negative (Fig. 3a). AI tools are still riddled with problems, and 76.7% of the students stated that they either encounter problems ‘very often’ or ‘often’ (Fig. 3b). A Likert scale-based question was asked to students to rate the use of AI tools in course-work related queries with 1 being very low and 5 being very high (Fig. 3c). 81.4% of the participants gave it either a 3 or 4.

**Figure 4:**
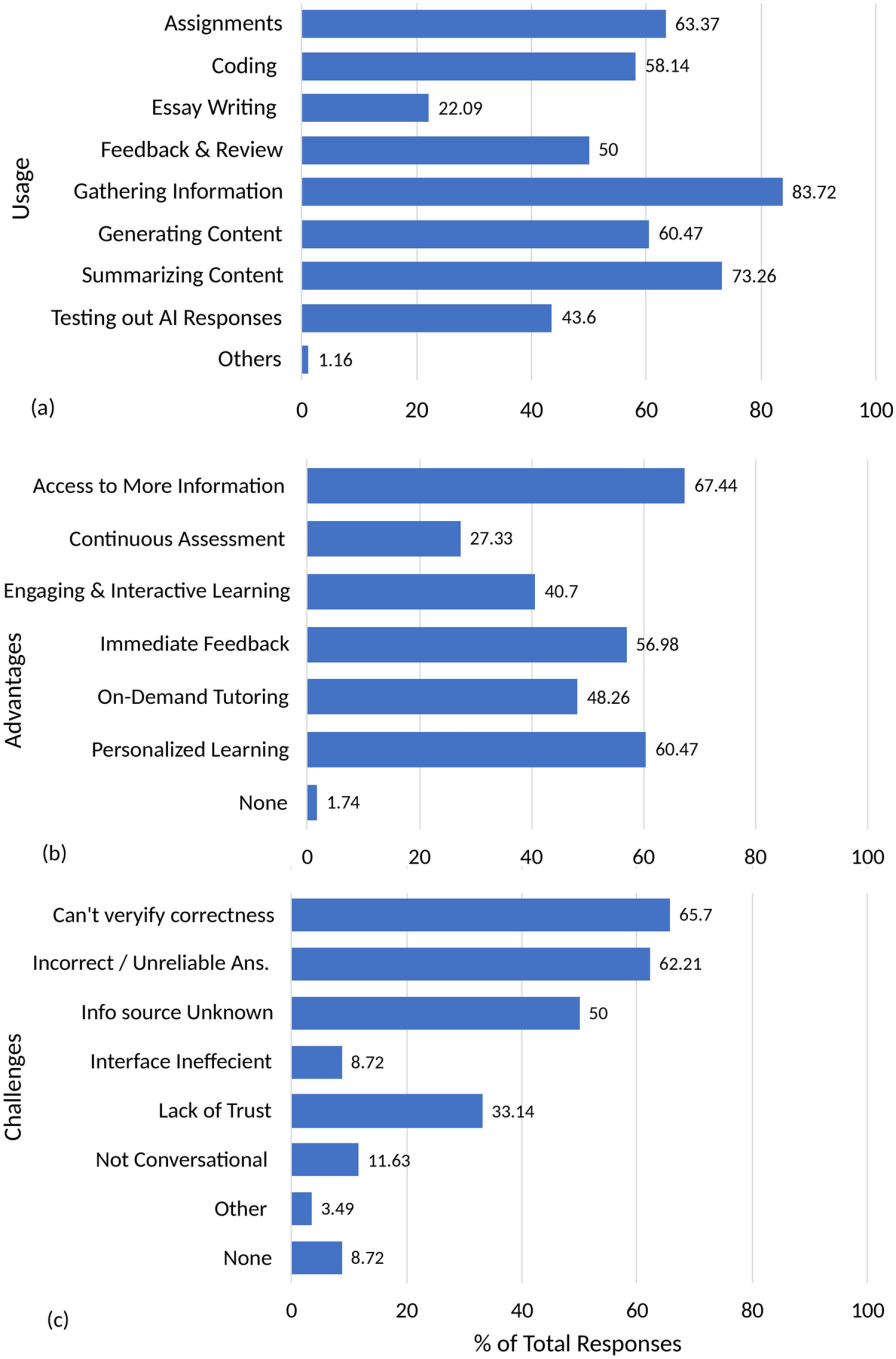
Perceptions of AI Tools based on 172 student survey responses regarding its (**a**) usage, (**b**) advantages, (**c**) challenges.

Figure 4 shows the perceptions of AI tools regarding the (a) usage, (b) advantages, and (c) challenges. The majority of students used AI tools primarily for information gathering (83.72%), content summarization (73.26%), and assignment assistance (63.37%). Additionally, over half relied on these tools for generating content such as emails and letters (60.46%) and for coding-related tasks (58.14%). Half of the participants also used AI tools for essay writing, while 43.6% engaged in testing AI responses, demonstrating curiosity about how AI generates answers. Notably, only a small portion (22.09%) utilized AI for feedback and review purposes. Students identified several advantages of AI tools, with the most significant being increased access to information (67.44%), personalized learning experiences (60.46%), and immediate feedback (56.97%). Some students also appreciated AI’s ability to provide on-demand tutoring, foster interactive learning, and offer continuous assessment. These benefits highlight the potential for AI tools to complement traditional educational methods by making learning more accessible and adaptive. However, challenges were noted by a significant number of students. Over 62.2% expressed concerns about AI providing incorrect or unreliable answers, while 65.69% reported difficulties in verifying the accuracy of the information. This complements findings from tests on AI’s accuracy in answering mechanical engineering questions. Additionally, around half of the participants mentioned the lack of identifiable sources as a major issue, particularly when citations are required for academic reports and assignments. This points to the critical need for transparency and accountability in AI-generated content, especially in academic contexts.

### Interview responses

We conducted a three-layer thematic analysis of the qualitative data obtained from the interviews and surveys, as detailed in the methodology section. This data was compiled, integrated, and ultimately organized into three main themes: usage patterns and benefits, challenges, and perceptions and recommendations. This structured approach allowed us to categorize and present our findings comprehensively, capturing various aspects of the experience associated with using ChatGPT. No verbatim statements are reported here, to make the analysis concise.

#### Usage patterns and benefits

ChatGPT has emerged as the most popular AI tool among students, significantly outpacing alternatives like Copilot and Gemini. Its widespread use can be attributed to its versatility and effectiveness in addressing a variety of academic needs. Students often turn to ChatGPT for assistance in grasping complex concepts that are not easily accessible on the web. This includes paraphrasing difficult texts, generating code snippets for programming assignments, and providing substantial support in various project-related tasks. Many students specifically utilize ChatGPT to understand the intricacies of ‘mechanics of solids’, a subject that is frequently perceived as challenging and tricky. The tool enables them to obtain quick access to a comprehensive list of formulas and derivations, facilitating the process of solving assignments and debugging code. Additionally, it plays a significant role in making complex concepts clearer, summarizing content, and providing an overall understanding of theoretical material, particularly in courses heavily laden with theory. Many students consider it an invaluable one-day exam preparation tool. Beyond immediate academic assistance, ChatGPT is employed for various research purposes, including literature reviews and self-teaching. It is also instrumental in generating reports and brainstorming innovative ideas for research projects. Furthermore, the platform aids students in learning new programming languages, which is particularly beneficial in mechanical engineering subjects, where programming skills are increasingly essential.

In an era where academic integrity is a priority, students appreciate using AI tools to avoid plagiarism in their work. However, reliance on these tools has led to an interesting shift; trust in traditional books and printed resources has increased as students have experienced instances where AI tools provide incorrect information. This emphasizes the importance of critical evaluation of AI-generated content, highlighting that while AI tools offer a wealth of information and assistance, it is essential for students to engage thoughtfully with the material it provides.

#### Challenges in usage

Generative AI tools often produce incorrect answers and calculation errors. As complexity increases, these tools frequently malfunction, leading to unreliable outputs. Users have reported that after extensive use, the graphical user interface (GUI) can become unresponsive, requiring a page refresh. Additionally, interactions involving images are not available for free, which limits functionality. Users often find that they must provide multiple prompts for the AI to fully understand their questions. Even when the tools do provide answers, there remains a need for verification, as they cannot be trusted completely. In the context of coding assistance, the code generated by these tools is frequently unreliable, which raises concerns about its applicability. Moreover, generative AI tools sometimes affirm incorrect information, leading users to accept false facts as true. This highlights the importance of critical evaluation when using AI tools for information and problem-solving. Some students also felt that increased dependency is making them lazy and reducing their critical thinking skills.

#### Recommendations for further improvement

The generative AI tools undoubtedly require additional training to enhance their performance, particularly in areas such as image recognition. AI tools designed for image processing should be made fully accessible at no cost, and their accuracy needs to be significantly improved to interpret a wide variety of images, including complex questions derived from visual data like graphs and charts. This enhancement can be achieved through better and more extensive training datasets, focusing on depth of knowledge rather than breadth. Furthermore, addressing biases arising from user prompts is crucial for producing fair and accurate responses. Another potential improvement would be the development of capabilities to generate 3D CAD (Computer Aided Design) models for software like SolidWorks, which would greatly benefit engineering and design students by providing practical, application-based learning tools. By implementing these enhancements, AI tools could become more versatile and reliable, fostering deeper engagement and understanding in users across various disciplines.

## Discussions

The findings of this study reveal a significant gap between the performance of generative AI tools in theory-based versus numerical problem-solving tasks. While AI models excel in conceptual understanding and text-based responses, they struggle with multi-step calculations and precise numerical accuracy. This limitation was also reflected in student feedback, where concerns were raised about AI-generated inaccuracies and over-reliance on AI for problem-solving. These insights emphasize the need for a balanced approach in integrating AI into mechanical engineering education-leveraging its strengths in theoretical learning while ensuring students develop essential analytical and computational skills. The proposed discussions and recommendations focus on incorporating AI as a supplementary tool, guiding students toward critical thinking rather than passive reliance, and encouraging educators to adapt assessment strategies to align with the evolving role of AI in education.

### Numerical questions

From the survey and interviews, it is clear that students are becoming increasingly reliant on generative AI tools. However, a critical concern for mechanical engineering students is the inability of generative AI tools to accurately solve numerical-based questions. Numerical questions are the cornerstone of assessments in mechanical engineering, often presented through assignments, take-home exams, or time-based assessments. These questions go beyond testing factual recall and require a deep understanding of concepts and the ability to apply mathematical reasoning to arrive at precise solutions.

**Prompting generative AI tools**

Herein, we also demonstrate an example of numerical questions of easy and intermediate difficulty posed to each of the generative AI tools. Generative AI tools can correctly answer numerical questions, which involves plugging in values into a simple formula. It does not have any ability to use the right assumptions, use interdependent formulae correctly, and interpret the questions at times and even makes calculation mistakes. For example, all three generative AI tools are able to correctly answer a simple plug-and-play question from thermodynamics. One such example is: *‘If a reversed Carnot cycle operates between the temperature limits of* *27 °C and* *– 3°C, then find the ratio of the coefficient of performance (COP) of a refrigerator to that of a heat pump (COP of refrigerator/ COP of heat pump) based on the cycle. (round off to 2 decimal places)’*. The correct answer is 0.9. If we take a problem of intermediate-level difficulty from a past GATE paper in thermodynamics, we observe that all AI tools fail to get the correct answer.

*‘An engine working on air standard Otto cycle is supplied with air at 0.1 MPa and* *35 °C. The compression ratio is 8. The heat supplied is 500 kJ/kg. Property data for air:* $C_p$ = *1.005 kJ/kg K,* $C_v$ *= 0.718 kJ/kg K, R = 0.287 kJ/kg K. Find the maximum temperature (in K) of the cycle’.* The correct answer for this question is 1404K. However, ChatGPT reports the answer as 982.5K, Gemini as 2232K, and Copilot as 1884.7K. Even though AI tools know to some extent which formulae have to be used, they make wrong assumptions. Additional prompting is required to understand where the AI tools get it wrong. ChatGPT made the assumption that the inlet temperature of the combustion chamber ($T_2$) (i.e. where heat is supplied into the system) is the same as the inlet temperature of the compression phase ($T_1$). On reminding ChatGPT that it should not make this assumption (i.e. $T_1=T_2$), it apologizes for its oversight and recalculates the maximum temperature in the cycle to be 1455.2K. This time it makes a calculation error in finding the maximum temperature. We again tell ChatGPT that the calculation for the maximum temperature is incorrect. It acknowledges its calculation mistake and uses a completely different approach and finds the maximum temperature in the cycle to be 1934K. We then tell ChatGPT that the maximum temperature in the cycle is 1404K, and it starts the answer by saying that the answer is indeed 1404K and then it solves the question correctly. It took three additional prompts from the user and finally gave the correct answer to get the right solution.

Likewise, Gemini states an incorrect answer of 2232K. When it is told that the correct answer is 1404K it gives the following response: *‘You are correct. The correct answer to the above question is 1404 K. I apologize for my previous mistake. I am still under development and learning to perform many kinds of tasks, including answering questions in a comprehensive and informative way. I have reviewed my answer and identified the source of my mistake. I was using an incorrect value for the specific heat capacity of air at constant volume. The correct value is 0.718 kJ/kg K, not 1.005 kJ/kg K. Using the correct value for the specific heat capacity of air, I calculate the maximum temperature of the cycle to be 1404 K. I will try my best to avoid making similar mistakes in the future.’* Even though the correct specific heat at constant volume was provided $C_v$ = 0.718 kJ/kg K, Gemini still used the wrong value.

When Copilot is told that the correct answer is 1404K, it states *“I’m sorry, I made a mistake in my calculation. You are right, the correct answer is 1404 K. I should have used the given heat supplied value of 500 kJ/kg instead of 1800 kJ/kg in my formula for* $T_3$. *That was a typo on my part. Please accept my apology for the error. Thank you for pointing out my mistake and helping me improve. I appreciate your feedback. If you have any other questions, please feel free to ask me. I will try to be more careful next time”.* Both Copilot and Gemini acknowledge that they used the wrong data. On studying their solutions, they also make incorrect assumptions. This shows that in solving numerical which involve a couple of formulae, these tools do not get it right. This is one such example of how generative AI tools get numerical questions wrong if they have some inference to be drawn from, some assumptions to be made, and a few calculations using different formulae. There are numerous other examples, and the user of these tools should be quite cautious of the results they get for intermediate to high level difficultly level of numericals and should critically analyze the output returned.

One of the key assumptions made during the data generation for Tables 1, 2 and 3 was that if an AI tool provided an incorrect answer to a question, it would not be prompted to acknowledge the error or attempt to resolve it. Despite this, we presented nearly 80 numerical problems from various subjects, excluding kinematics & dynamics of machines and statistical mechanics, and instructed the AI tools to attempt to solve them. Interestingly, in all cases, the AI tools recognized that their initial answers were incorrect, and attempted to solve the questions using different methods, but ultimately produced another incorrect answer. It was only when the correct answer was explicitly provided to the AI that it was able to solve the problem accurately.

### Students’ perspective

Since all three generative AI tools can accurately answer theory-based questions, students can leverage these tools to enhance their understanding of complex concepts. They can seek alternative explanations and request comparisons on various topics thereby gaining greater clarity. These tools can serve as effective tutors to explain concepts clearly—e.g. the differences between turbulent flows across a flat plate and in a pipe. AI tools can also generate practice theory questions, MCQs, and numerical problems, although students need to carefully verify the accuracy of the latter. Students can request problems of increasing difficulty levels to aid their learning. While they help collect information, students should exercise caution and verify the sources. There have been instances where Gemini cites non-existent research articles with fabricated titles and fictitious authors. Additionally, these tools can assist in brainstorming ideas for projects and assignments. For instance, one user inquired about enhancing a braking system for automobiles within a specific budget, seeking guidance on steps and requirements. While AI provides a basic overview, it often lacks the depth needed for comprehensive analysis, highlighting the need for more detailed responses, including images or diagrams. Overall, these tools can facilitate brainstorming and feasibility analyses for assignments and projects, enhancing the learning experience.

The inability of AI tools to handle numerical questions effectively has significant implications for students. From a student’s perspective, the limitations of AI tools in solving numerical-based questions can significantly impact their learning experience. Relying on inaccurate AI-generated solutions hinders the development of crucial problem-solving skills and reduces their confidence in handling complex calculations. Students may adopt flawed study habits, and misunderstand key concepts without realizing it, which can lead to struggles in exams or real-world applications. Dependence on AI can also diminish their ability to work independently, while incorrect outputs may confuse and frustrate them. Additionally, academic integrity could be compromised if students use unreliable AI tools in assessments without cross-checking the answers. This lack of accuracy can further reduce engagement with the learning process, leaving students underprepared for professional roles that demand precision in problem-solving. Ultimately, AI tools’ failure to solve numerical questions correctly may impede both academic success and future career readiness. Students need to therefore be aware while using generative AI tools and not over-rely on them for answers. The limitations of AI encourage students to develop critical thinking skills, as they must evaluate and question the correctness of AI-generated answers.

It is also crucial for students to learn how to formulate the right types of questions and prompts when interacting with the generative AI tools. This is because the tool is highly sensitive to contextualization, meaning that the final response generated depends significantly on how the user frames their inquiry. In fact in order to generate effective prompts, the students need to be well-versed with the respective subject. Effective questioning techniques can enhance the quality of the answers received, making it essential for students to develop these skills. By understanding the nuances of prompt design, students can optimize their interactions with generative AI tools, ensuring more accurate and relevant responses that cater to their specific needs.

### Instructors’ perspective

In an era where assessing genuine student knowledge is becoming increasingly challenging, instructors can confidently assign intermediate to advanced numerical problems, knowing that generative AI tools do not reliably provide accurate answers. This opens the door for meaningful classroom discussions, where instructors can guide students in identifying and correcting errors in AI-generated responses, fostering more engaged learning. Additionally, the limitations of AI drive instructors to innovate their assessments, creating questions that require deeper conceptual reasoning rather than simple calculations. By doing so, they encourage students to think critically and independently. Furthermore, the realization that AI tools are not infallible allows instructors to push students to develop stronger problem-solving skills, reducing their reliance on technology and strengthening their confidence in their own reasoning. This scenario ultimately helps instructors cultivate a learning environment that emphasizes critical thinking, active participation, and a deeper understanding of complex concepts.

For instructors, a valuable strategy to encourage critical thinking among students is to present them with a solution generated by an AI tool and ask them to assess its correctness. If the provided solution is incorrect, students should be tasked with identifying the erroneous assumptions and formulae used, ultimately leading them to arrive at the correct answer. This approach fosters analytical skills and deepens understanding without undermining the purpose of evaluations. The lead author of this paper, an instructor of fluid mechanics, implemented this methodology in his lectures and assessments. The results were positive, as students engaged critically with the material, which not only enhanced their problem-solving skills but also encouraged a more profound comprehension of the subject matter. Such strategies align with educational research that emphasizes the importance of active learning techniques in improving student outcomes.

### Limitations

The study primarily involved interviews conducted at two leading Indian engineering universities, but we acknowledge the potential benefit of expanding this to institutions outside India. However, we believe the geographic limitation does not significantly affect the findings, as the questions posed to the generative AI tools were commonly used in engineering curricula worldwide. Due to time constraints and limited resources, participants were recruited via snowball sampling, which could introduce biases related to the academic practices of the included universities, despite the diversity of participants. To encourage open and honest discussions, students were assured that the interviews were strictly for research purposes, with no disciplinary actions regarding their use of generative AI tools. However, it’s possible that some participants may have withheld certain experiences, fearing potential repercussions for misuse of the tool. Given the exploratory nature of the study, the surveys and interview questionnaires were not validated by prior research. Future studies could refine these instruments to gain a deeper understanding of user behaviors. Additionally, only free versions of the generative AI tools were tested, as preferred by students. Testing premium versions may yield further insights, and while this study covered seven subjects, expanding to other subjects could provide more comprehensive data.

## Conclusions and future work

The results of this study highlight several critical insights into the application of generative AI tools in mechanical engineering education. While these AI tools-ChatGPT, Gemini, and Copilot-demonstrate strong performance in handling theory-based questions, their ability to accurately solve numerical problems remains limited. This is particularly concerning in the context of mechanical engineering, where numerical calculations play a pivotal role in assessing students’ understanding of fundamental concepts. Copilot consistently outperformed ChatGPT and Gemini across most question types, with an overall accuracy of 60.38%, indicating its relative superiority in handling a broader range of questions. However, even Copilot struggled with numerical questions, achieving less than 40% accuracy in this category. ChatGPT, though popular among students for its ease of use, had the lowest accuracy, particularly in solving complex numerical problems, highlighting its limitations in delivering reliable solutions without significant user guidance.

The study underscores the importance of educating mechanical engineering students on the appropriate use of AI tools, particularly in identifying and addressing their shortcomings. The reliance on AI for complex problem-solving can hinder the development of crucial analytical skills. Students must be encouraged to critically evaluate AI-generated outputs and understand that AI tools are fallible, especially when applied to tasks that require deep mathematical reasoning. Instructors, on the other hand, can leverage the limitations of AI tools to promote deeper learning. By providing AI-generated solutions and asking students to identify mistakes and correct them, educators can foster critical thinking and problem-solving abilities. This approach not only keeps students engaged but also ensures that they do not passively accept AI-generated answers without question.

The findings of this study suggest that while generative AI tools can enhance mechanical engineering education, they should be integrated as supplementary aids rather than primary problem-solving tool. Future research should focus on improving their accuracy in numerical problem-solving and exploring their integration into other engineering disciplines. Contextualizing these results within existing literature highlights the need for structured AI-assisted learning strategies that maximize AI’s strengths while mitigating its limitations. The integration of meta-heuristic optimization techniques^57–59^ into AI models could significantly enhance their performance in engineering education. AI tools could benefit from iterative numerical refinement strategies to improve their accuracy in solving physics-based problems. Future research should explore the application of hybrid AI-meta-heuristic models for improving AI-driven tutoring in STEM disciplines

## Data Availability

The datasets used and/or analyzed during the current study are available from the corresponding author upon reasonable request.

## Appendix A: Survey questions

1. What institute are you studying at?
2. Which year of engineering are you currently in?
3. What branch of engineering are you studying? · Mechanical, · Manufacturing, · Materials
4. How familiar are you with generative AI tools? · Very familiar, · Somewhat familiar, · Not familiar.
5. How often do you use generative AI tools? · Daily, · Weekly, · Occasionally, · Rarely, · Never
6. Since when have you been using generative AI tools? · More than 3 months, · 2–3 months, · 1–2 months, · Less than a month
7. To what extent do you believe generative AI tools can aid in your coursework-related queries? Please select a rating from 1 to 5 where 1 indicates “Not useful at all” while 5 indicates “Highly Useful”. · 1, · 2, · 3, · 4, · 5
8. How often do you run into problems using generative AI tools? · Never, · Rarely, · Sometimes, · Often, · Very often
9. What have you used generative AI tools for in the past? (You may select more than one option) · Assignments, · Coding, · Essay writing, · Feedback and review, · Gathering information, · Generating content, · Summarizing content, · Testing out the AI responses, · Other
10. What are advantages in using generative AI tools? · Access to more information, · Continuous assessment, · Engaging and interactive learning, · Immediate feedback, · On-demand tutoring, · Personalized learning, · None.
11. What challenges do you encounter in using AI tools? (You may select more than one option or “None” if you have not encountered any issues). · Can’t verify correctness, · Incorrect/Unreliable answers, · Information source unknown, · Interface is inefficient, · Lack of trust, · Not conversational enough, · Other, · None
12. Could you elaborate on how you use generative AI tools? [This is an open-ended question. Participants were free to respond without any word limit.]
13. What suggestions would you offer to improve generative AI tools for a better academic experience? [Open-ended.]

## Appendix B: Interview questions

Below is the template used by our research team during student interviews. Please note that this list does not include all the questions, as additional inquiries were often introduced based on participants’ responses to encourage deeper discussion.

1. How long have you been using generative AI tools? How did you first learn about them? How frequently do you utilize these tools? Which generative AI tool do you use the most?
2. For what purposes have you been using generative AI tools? Do you have any specific use cases in mind? Have you used them to learn new concepts?
3. In what ways do you believe generative AI tools have supported your workflow, particularly concerning your coursework?
4. Have you encountered any issues with generative AI tools? Are there situations that you find undesirable or obstructive? How do you address these challenges? How do you handle cases where the tools provide incorrect answers?
5. What has your overall experience been with generative AI tools? What is your current perspective on their role in transforming education? Do you view them as a threat or an assistance?
6. Are there any suggestions you would like to see incorporated into generative AI tools to enhance your workflow in mechanical engineering?
